# PD77 Review Of Multicriteria Decision Analysis-Like Processes For Prioritization Of Newborn Bloodspot Screening Conditions

**DOI:** 10.1017/S0266462324003325

**Published:** 2025-01-07

**Authors:** Eanan Finnegan, Arielle Maher, Karen Jordan, Helen O’Donnell, Laura Comber, Susan Spillane, Patricia Harrington, Máirín Ryan

## Abstract

**Introduction:**

Newborn bloodspot screening (NBS) is used to identify rare, serious health conditions. The choice of conditions to screen for can be contentious, with no internationally agreed panel. Multicriteria decision analysis (MCDA), involving a priori identification and weighting of criteria, has been proposed as an approach to assess and rank conditions for inclusion in newborn screening panels or for further assessment.

**Methods:**

The aim of this review was to summarize existing MCDA-like processes used internationally in the context of NBS conditions. Publications were identified using a scoping methodology including electronic database and gray literature searches. Information on the methodology used to derive criteria and weightings, the criteria used, the scoring systems, and the weightings were extracted. The results were synthesized narratively.

**Results:**

Five publications reporting on the use of MCDA or MCDA-like processes in the context of NBS were identified. In three publications, the aim of the MCDA processes was to select conditions for inclusion in an NBS panel. Two referred to the potential use of MCDA to inform prioritization of candidate conditions for future evaluation. While the criteria used were broadly consistent across studies, assigned weightings varied. Key challenges noted in the studies in relation to applying MCDA included the subjectivity associated with the choice and weighting of criteria, the absence of data to inform criteria, and its low discriminatory power.

**Conclusions:**

Choosing criteria and weightings for MCDA processes regarding NBS can be a highly deliberative process that is intrinsically linked to the values and perspectives of those involved. Given the lack of international consensus on criteria and weightings, MCDA processes to aid decision-making would likely require a de novo tool or careful adaptation of an existing tool to reflect local contexts.

